# Influence of a sodium-saccharin sweetener on the rumen content and rumen epithelium microbiota in dairy cattle during heat stress

**DOI:** 10.1093/jas/skac403

**Published:** 2022-12-13

**Authors:** Lucas R Koester, Kris Hayman, Chiron J Anderson, Bienvenido W Tibbs-Cortes, Karrie M Daniels, Faith M Seggerman, Patrick J Gorden, Mark Lyte, Stephan Schmitz-Esser

**Affiliations:** Department of Veterinary Microbiology and Preventive Medicine, Iowa State University, Ames, IA 50011, USA; Interdepartmental Microbiology Graduate Program, Iowa State University, Ames, IA 50011, USA; Department of Veterinary Diagnostic and Production Animal Medicine, Iowa State University, Ames, IA 50011, USA; Interdepartmental Microbiology Graduate Program, Iowa State University, Ames, IA 50011, USA; Department of Animal Science, Iowa State University, Ames, IA 50011, USA; Interdepartmental Microbiology Graduate Program, Iowa State University, Ames, IA 50011, USA; Department of Animal Science, Iowa State University, Ames, IA 50011, USA; Department of Veterinary Microbiology and Preventive Medicine, Iowa State University, Ames, IA 50011, USA; Interdepartmental Microbiology Graduate Program, Iowa State University, Ames, IA 50011, USA; Department of Animal Science, Iowa State University, Ames, IA 50011, USA; Department of Veterinary Diagnostic and Production Animal Medicine, Iowa State University, Ames, IA 50011, USA; Department of Veterinary Microbiology and Preventive Medicine, Iowa State University, Ames, IA 50011, USA; Interdepartmental Microbiology Graduate Program, Iowa State University, Ames, IA 50011, USA; Department of Animal Science, Iowa State University, Ames, IA 50011, USA

**Keywords:** dairy cattle, heat stress, rumen epithelium, rumen microbiota, saccharin

## Abstract

The effect of a saccharin-based artificial sweetener was tested on animal performance measures and on the microbial communities associated with the rumen content and with the rumen epithelium during heat stress. Ten cannulated Holstein-Friesian milking dairy cattle were supplemented with 2 g of saccharin-based sweetener per day, top-dressed into individual feeders for a 7-day adaptation period followed by a 14-day heat stress period. A control group of ten additional cows subjected to the same environmental conditions but not supplemented with sweetener were included for comparison. 16S rRNA gene amplicon sequencing was performed on rumen content and rumen epithelium samples from all animals, and comparisons of rumen content microbiota and rumen epithelial microbiota were made between supplemented and control populations. Supplementation of the saccharin-based sweetener did not affect the rumen content microbiota, but differences in the rumen epithelial microbiota beta-diversity (PERMANOVA, *P* = 0.003, *R*^2^ = 0.12) and alpha-diversity (Chao species richness, *P* = 0.06 and Shannon diversity, *P* = 0.034) were detected between the supplemented and control experimental groups. Despite the changes detected in the microbial community, animal performance metrics including feed intake, milk yield, and short-chain fatty acid (acetic, propionic, and butyric acid) concentrations were not different between experimental groups. Thus, under the conditions applied, supplementation with a saccharin-based sweetener does not appear to affect animal performance under heat stress. Additionally, we detected differences in the rumen epithelial microbiota due to heat stress when comparing initial, prestressed microbial communities to the communities after heat stress. Importantly, the changes occurring in the rumen epithelial microbiota may have implications on barrier integrity, oxygen scavenging, and urease activity. This research adds insight into the impact of saccharin-based sweeteners on the rumen microbiota and the responsivity of the rumen epithelial microbiota to different stimuli, providing novel hypotheses for future research.

## Teaser

Heat stress has been shown to impact dairy cattle rumen microbial communities, which are essential for host health and performance. A saccharin-based palatability enhancer modified rumen microbial communities during a heat stress challenge, resulting in whole community differences and a decrease in overall community diversity.

## Introduction

The effects of increasing global temperatures are of major concern to livestock production. As ambient temperatures increase, the ability of animals to maintain thermal homeostasis through sensible (conduction, convection, and radiation) and latent (evaporation through sweating or panting) heat loss decreases (Nonaka et al., 2008; Brown-Brandl, 2018). When experiencing heat stress, cattle reduce their dry matter intake (DMI) thereby reducing gross energy intake. This energy loss is further compounded by an increased energy expenditure via heat dissipation behaviors (increased respiration and heart rates) (Silanikove, 2000; Brown-Brandl, 2018). This reduction in net available energy translates to a reduction in growth rate and milk yield (Rhoads et al., 2009; Wheelock et al., 2010; Gao et al., 2017; Brown-Brandl, 2018; Menta et al., 2022). Additionally, chronic heat stress has been shown to reduce reproductive efficiency, with a delay in puberty and time to first calving in heifers and increased incidence of retained placenta and metritis infections in cows (Menta et al., 2022). Finally, under extremely high temperature conditions, livestock animals may die as a result of severe heat stress as reported during recent heat waves (Bishop-Williams et al., 2015).

Typically, the temperature humidity index (THI), a measurement accounting for the ambient temperature and humidity, is used to determine heat stress in cattle. Early research indicated a THI of 72 marked the beginning of heat stress, but recently new evidence has shown that a THI as low as 68 led to reduced milk yield and other economically important performance measures in Holstein dairy cattle (Armstrong, 1994). Economic losses due to heat stress in cattle are mainly driven by decreases in milk or meat yield, increases in mortality, and decreased reproductive success. St-Pierre et al. (2003) estimated that in the 21 states in the United States with moderate to high maximal THI values (<80), a single dairy cow can be expected to consume 511 lbs less feed and produce 1,032 lbs less milk annually. Overall, St-Pierre et al. (2003) estimated heat stress results in $897 to $1,500 million losses to the United States dairy industry, though the actual losses may be higher due to variables affected by heat stress which were not accounted for. One important and understudied effect of heat stress is its impact on the microbial communities that reside within the gastrointestinal (GI) tract of the ruminant host.

Rumen microbial communities are essential to host health and performance as microbial fermentation of feed provides key metabolic products for the host animal (Russell and Hespell, 1981; Bergman, 1990; Hess et al., 2011; Morgavi et al., 2013; Weimer, 2015; Paz et al., 2018; Xue et al., 2020; Zhou et al., 2021). Rumen microorganisms produce nutritionally important fermentation products such as short chain fatty acids (SCFA) and vitamins via the breakdown of cellulose, hemicellulose, pectin, and other ingested feed (Bergman, 1990; Seck et al., 2017; Averianova et al., 2020; Beckett et al., 2021). These microbial metabolites produced in the rumen are absorbed directly by the host through the rumen epithelial tissue. Rumen microorganisms have been characterized into three main groups: rumen solid-associated microorganisms, rumen fluid microorganisms, and rumen epithelial microbiota. The first two groups are collectively denoted as rumen content microbiota (McCowan et al., 1978; Cheng et al., 1979; Zhou et al., 2021; Na and Guan, 2022). Several studies have analyzed the community structure and function of the rumen content microbiota, emphasizing its importance in feed efficiency and host performance (Hackmann and Spain, 2010; Henderson et al., 2015; Gruninger et al., 2019; O’Hara et al., 2020). Recent studies, including two meta-analyses of 16S rRNA gene sequencing data, have contributed greatly to the taxonomic analysis of the rumen epithelial microbiota, but the functional capacity of these organisms remains largely unknown (Mann et al., 2018; Anderson et al., 2021; Pacífico et al., 2021; Schmitz-Esser, 2021).

There is a growing interest in how heat stress affects rumen microbial communities, and recent research has demonstrated that the rumen content microbiota is directly affected by heat stress (Zhao et al., 2019; Kim et al., 2022; Wang et al., 2022). However, none of these studies have analyzed the rumen epithelial microbiota, and the effects of heat stress on the rumen epithelial microbiota remain unknown. A major consequence of heat stress is the reduction of feed intake, ruminal motility, and passage rate, resulting in lower available substrate for the microbial communities of the rumen. Additionally, changes in host physiology as a result of heat stress may have effects on microbial population dynamics and metabolic outcomes. These consequences would most acutely be felt in animals that rely heavily on their microbiota, such as ruminants.

One way to address the reduction in performance due to heat stress conditions is to encourage feed intake. Different palatability enhancers have been suggested for improving livestock feed intake, including sodium-saccharin based sweeteners, and have been marketed for many different livestock species including swine, sheep, and beef and dairy cattle. Previous research demonstrated that when beef cattle experiencing the stress of acclimating to a feedlot setting were fed 200 g of the saccharin-based sweetenerSucram (Pancosma/ADM)/ton of dry matter (DM), they had a numerically higher average daily gain (ADG) than beef cattle that did not receive Sucram (Ponce et al., 2014). Other experiments testing the effect of continuously supplemented Sucram on DMI and ADG found that both measures tended to be greater in feedlot cattle from day 29 to 56 of the receiving period, and there was also a tendency for DMI to be higher in cattle supplemented with Sucram during the finishing period (McMeniman et al., 2006). Because saccharin-based sweeteners appear to have a positive effect on cattle under these stressors, we hypothesize that saccharin-based sweeteners may also positively affect performance during heat stress.

In this study, we tested the effects of a saccharin-based sweetener on different animal performance measures and both rumen content microbiota and rumen epithelial microbiota during heat stress. Our hypothesis was that the supplementation of a saccharin-based sweetener would help maintain feed intake, thereby reducing the impact of heat stress on the animals, their rumen microbiota, and the microbial metabolic fermentation products.

## Methods

### Ethics statement

All animal procedures in this study were conducted under approval of the Animal Care and Use Committee at Iowa State University (ISU) (IACUC-19-225).

### Animal trial

To study the effect of the artificial sweetener Sucram (Pancosma S.A./ADM Groups, Rolle, Switzerland) on rumen microbial communities during chronic heat stress, rumen epithelium and content samples were taken from twenty rumen-cannulated, lactating Holstein-Friesian dairy cows of differing parity. The twenty cows were divided into two replicates of ten, and these were further divided into five treatment and five control cows. Replicate 1 cows were all first parity, ranging in body weight from 1,140 to 1,385 lbs and between 62 and 122 d in milk, and were producing 62–87 lbs of milk per day. Replicate 2 cows were from parities 1–3, ranging in body weight from 1,170 to 1,405 lbs and between 31 and 85 d in milk, and were producing 90–123 lbs of milk per day. Cows within the treatment group received 2 g Sucram a day, manually mixed into the feed as per feeding protocols provided by Pancosma. All animals were housed at the Iowa State University (ISU) dairy teaching farm under the same conditions and fed the same diets comprised of ground corn, soybean meal, dry corn gluten, corn silage, baleage, and alfalfa hay (50.9% dry matter (DM), 16.8 % crude protein (CP), and 54.63% neutral detergent fiber (NDF)) (Table 1). Cows were randomly assigned to individual pens with access to their own feeder. All cows had ad libitum access to water.

**Table 1.** Dietary composition

Ingredients	Diet (% DM)
Corn silage	34.6
Master mix^1^	52
Alfalfa hay	13.4
Analyzed components	
Crude protein,^2^ %	16.8
NDFom,^2^ %	25.1
Ether extract,^2^ %	4.6
NEI,^2^ Mcal/kg DM	1.7
DM,^2^ %	50.9

^1^Mastermix includes Ground Corn (53%), Dry Corn Gluten (8.6%), Lactation Mix MPPPNT (16%), Soy Plus (5%), Straw Shriver (3.4%), and soybean meal (13.7%).

^2^Chemical analysis was completed by Dairyland Labs, MN, USA.

**Figure 1.**
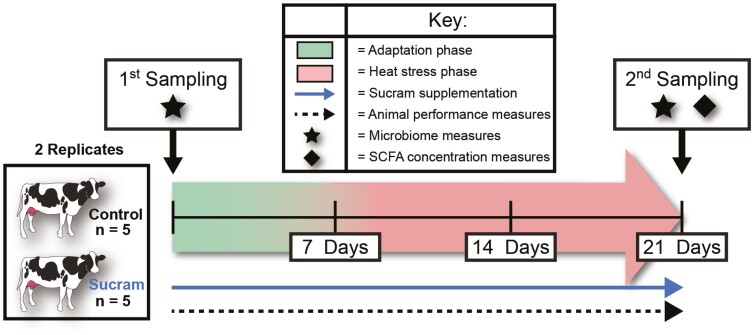
Experimental trial design describing trial timeline for each replicate. Feed intake measures were taken once per day, whereas other animal performance measures (milk yield, rectal temperatures and ambient THI) were measured multiple times a day and averaged. Heat stress was maintained beginning at 0800 h and ending at 1800 h each day during the heat stress period.

Each replicate was subject to a 7-day adaptation period, allowing animals to adjust to new diets and a different environment. Following the adaptation period, a 14-day heat stress period was applied within a closed barn using heaters, maintaining a cyclic heat stress environment (average THI of 80, never exceeding 95) beginning at 0800 h and ending at 1800 h each day. After 1800 h, barn screens were opened and heat stress was reprieved until 0800 h the following day. During each period, a rectal thermometer (Model#: 000.200000.2, Midwest Veterinary Supply, Des Moines, IA, USA) was used to obtain rectal temperatures from all animals at 0600 h, 1200 h, and 1800 h. Body weights were recorded weekly prior to the AM milking. Feed intake (FI) (lbs fed − lbs remaining) was recorded daily for each cow. Cows were milked twice daily (0500 h and 1700 h), with yields (milk weight) recorded at each milking utilizing an automated system (Boumatic SmartDairy (2060) system). Figure 1 provides a graphical representation of the experimental design and timeline.

### Sample collection and DNA extraction for 16S rRNA gene amplicon sequencing

A total of 40 rumen content and 40 rumen epithelial samples were collected before the adaptation period and after the heat stress period (Figure 1). Rumen content was collected by removing submerged fibrous material from the rumen through the fistula with sterile gloves and compressing it to collect approximately 40 mL of rumen fluid in sterile 50 mL conical tubes. The conical tubes were then placed directly on dry ice and allowed to freeze on site before transport to the laboratory. Rumen epithelium biopsies were collected using Chevalier Jackson forceps from two locations (separated vertically by 40 cm) in the dorsal rumen sac. After removal, rumen epithelial tissue samples were briefly rinsed in sterile 1x PBS, immediately snap frozen in liquid nitrogen on site, and placed in sterile, 2 ml screw-top centrifuge tubes and stored on dry ice. All rumen content and epithelial samples were returned to the lab directly and stored at −80 °C after collection.

For DNA isolation, rumen content and epithelium samples were thawed, and genomic DNA was extracted from approximately 0.2 g of rumen content sample and approximately 0.1 g of rumen epithelial sample using the Qiagen DNeasy Powerlyzer Powersoil kit following the manufacturer’s instructions. Mechanical cell lysis was performed using a Fischer Scientific Beadmill 24, and DNA concentrations were determined using a Qubit 3 fluorometer (Invitrogen, Carlsbad, CA, USA).

After extraction, DNA concentrations were adjusted to 25–85 ng/µl and sent to the ISU DNA facility for sequencing using the Illumina MiSeq platform (Illumina, San Diego, CA, USA). Briefly, the genomic DNA from each sample was amplified using Platinum Taq DNA Polymerase (Thermo Fisher Scientific, Waltham, MA, USA) with one replicate per sample using universal 16S rRNA gene bacterial primers (515F [5ʹ-GTGYCAGCMGCCGCGGTAA-3ʹ] and 806R [5ʹ-GGACTACNVGGGTWTCTAAT-3ʹ]), amplifying the variable region V4 as previously described (Kozich et al., 2013). All samples underwent PCR with an initial denaturation step at 94 °C for 3 min, followed by 45 s of denaturing at 94 °C, 20 s of annealing at 50 °C, and 90 s of extension at 72 °C. This was repeated for 35 total PCR cycles and finished with a 10 min extension at 72 °C. All PCR products were then purified with the QIAquick 96 PCR Purification Kit (Qiagen, Hilden, Germany) according to the manufacturer’s instructions. PCR bar-coded amplicons were mixed at equal molar ratios and used for Illumina MiSeq paired-end sequencing with 250 bp read length and cluster generation with 10% PhiX control DNA on an Illumina MiSeq platform at the ISU DNA facility. Barcodes and primers within each read were removed by the facility.

### Sequence analysis

Sequence analysis was performed using the Mothur V1.43.0 software following the Mothur MiSeq Standard Operating Procedure (Schloss et al., 2009). Paired-end reads were merged, creating contiguous sequences (contigs), and quality filtered using the “make.contigs” command in mothur. All contigs were then filtered using a minimum read length of 250 bp, a zero ambiguities threshold, and a maximum homopolymer length of eight bases. Possible chimeric sequences were removed using the “chimera.vsearch” command in mothur using the SILVA.gold reference database provided by the mothur website. For alignment and taxonomic classification of operational taxonomic units (OTUs), the SILVA SSU NR reference database (V138) provided by the mothur website was used. Sequences were clustered into *de novo* OTUs with a cutoff of 99% 16S rRNA sequence similarity (=0.01 distance). To reduce the number of spurious OTUs, all OTUs represented by less than 10 reads were removed prior to subsequent analysis. As two rumen epithelial samples were taken per animal per time point, these two samples were merged in silico to account for microbial differences based on biogeography within the rumen, resulting in 40 total rumen epithelial microbiota samples. Analyses of the rumen content microbiota was conducted separately from rumen epithelial microbiota samples to maintain resolution of the most abundant OTUs.

### Short-chain fatty acid analysis

The acetic, butyric, and propionic acid concentrations of rumen content from each animal at the final time point were quantified using the method described in De Baere et al.(2013). Briefly, rumen content material was centrifuged at 4,300 × *g* for 10 min. 900 µl of supernatant was transferred to a new tube and acidified with 100 µl of 2N HClO_4_. Acidified fluid samples were incubated at room temperature for 10 min and 5 ml of (C_2_H_5_)_2_O (diethyl ether) was then added to each sample. Samples were then tumble rotated for 20 min prior to an additional centrifugation step of 4,300 × g for 2 min. The ether layer was then transferred to a new tube, and 800 µl of 2N NaOH was added. Once again, the samples were tumble rotated for 20 min prior to centrifugation step of 4,300 × *g* for 2 min. Finally, the ether layer was removed and discarded, and 800 µl of remaining solution was acidified with 10N HCl prior to ultrahigh-performance liquid chromatography with UV/VIS detection (UHPLC-UV) analysis.

UHPLC-UV analysis of each sample was conducted using a Dionex UltiMate 3000 autosampler (WPS-3000), a Dionex UltiMate 3000 pump (ISO-3100BM), and a Dionex UltiMate 3000 UV/VIS detector (VWD-3400) (Thermo Fisher Scientific, Sunnyvale, CA). Buffered 10% acetonitrile (Catalog #: NC9777698, Thermo Fisher Scientific) was used as the mobile phase, and the flow rate was 0.6 mL/min on a 150 mm length, 3 mm internal diameter, and 3 μm particle size Hypersil BDS C18 column (Catalog #: 28,103-153030, Thermo Fisher Scientific). All samples were kept at 4 °C in the autosampler prior to injection. The UV detection was set to 210 nm. Data were analyzed using the Chromeleon software package (version 7.2, Thermo Fisher Scientific), and acetic acid, butyric acid, and propionic acid were detected; identification was confirmed using the relative retention time of the corresponding analytical standard from Millipore-Sigma (CAS Numbers: 79-09-4, 64-19-7, 107-92-6; Catalog #: AC149300010, A35-500, 18-611-912).

### Statistics

Comparisons in animal performance between Sucram and control animal groups, as well as Bray-Curtis dissimilarity between experimental groups, were made according to the following statistical model:


$$\begin{array}{l} Y_{ijklm}=\;\;\;\mu +S_{i\;\;\;}+r_{j\;\;\;}+S_{i\;\;\;}r_{j\;\;\;}+C_{k\;\;\;}+A_{\;\;\;l}+e_{ijklm} \end{array}$$ (1)

Where $Y_{ijklm}$ is the observed value for kth experimental unit within the ith level of Sucram status (Sucram or control) at the jth replicate (R1 vs. R2) accounting for the repeated effects of the kth cow and the baseline value included as a covariate ($A_{\;\;\;l}$); μ is the general mean; $S_{i\;\;\;}$ is the fixed effect of the ith treatment (i = Sucram or control); $r_{j}$ is the fixed effect of the jth replicate (j = R1 vs. R2); $S_{i\;\;\;}r_{j}$ is the subsequent interaction of Sucram status and replicate; $C_{k\;\;\;}$ is the repeated effect associated with each cow; $A_{\;\;\;l}$ is the average value for each cow, which was used as a baseline and treated as a covariate within the model; $e_{ijklm}$ is the associated variance as described by the model for $Y_{ijklm}$ (*k* = *k* through 20). Compound symmetry (CS) or heterogeneous first-order autoregressive structure AR (1) covariance structures were selected for each test based on lowest BIC values (lowest values represent best fit). Animal ID was included as a repeated variable within Sucram status.

A reduced model was used to compare the effects of Sucram on microbial community alpha and beta diversity, as we only had before (day 0) and after heat stress (day 21) samples. Thus, we removed the Animal ID repeated measure associated with each cow. The initial community measures were treated as the adaptation covariate within the following model:


$$\begin{array}{l} Y_{ijlm}=\;\;\;\mu +S_{i\;\;\;}+r_{j\;\;\;}+S_{i\;\;\;}r_{j\;\;\;}+A_{\;\;\;l}+e_{ijlm} \end{array}$$ (2)

Finally, as we only measured SCFA concentrations after heat stress, we used a model that excluded the adaptation covariate.


$$\begin{array}{l} Y_{ijm}=\;\;\;\mu +S_{i\;\;\;}+r_{j\;\;\;}+S_{i\;\;\;}r_{j\;\;\;}+e_{ijm} \end{array}$$ (3)

Measurements of Chao species richness, Shannon Diversity, and Simpson evenness, as well as animal performance measures (FI and MW) and SCFA (acetate, butyrate, and propionate) concentrations were compared between experimental groups. Least square means (LSmeans) of each measurement were calculated for Sucram or control groups and analyzed using the PROC MIXED procedure within SAS (Version 9.4, SAS Inst., Cary, NC) according to the described model (Eq. (2)). Pairwise comparisons were corrected for multiple testing using Tukey’s Honest Significant Difference test, and treatment differences were considered significant if *q* values (*q*) were < 0.05 (Tukey, 1949).

Bray-Curtis dissimilarity comparisons between experimental groups, which included the adaptation time point as a comparison group, were analyzed using PERMANOVA and the variation BetaDisp command (BetaDispersr) provided within the VEGAN (v2.5-5; (Oksanen et al., 2019)) package within R (v4.1.0) according to the same model (Eq. 1). All pairwise contrasts between levels within the independent variables were made using pairwiseAdonis (v0.4; (Martinez Arbizu, 2020)) within R and were corrected for multiple testing using Bonferroni’s correction (Dunn, 1961). Overall variation between bacterial communities (beta diversity) was visualized using principle coordinate analysis (PCoA). This information was generated using a combination of Phyloseq (v1.34.0; (McMurdie and Holmes, 2013)), and VEGAN (v2.5-5; (Oksanen et al., 2019)). All plotting was completed using the ggplot2 (v2_3.1.1, (Wickham, 2016)) graphing package within R.

Finally, the absolute abundances of the 100 most abundant OTUs and all phyla among papillae and content samples were analyzed using a negative binomial distribution in the GLIMMIX procedure of SAS according to the models described above (Eq. (2)). All count data were offset by the total library count for a given sample. Corresponding *P*-values were corrected for false discovery rates using the FDR correction of the MULTITEST procedure within SAS. For all comparisons, *P* (or *q*) values were considered significant if <0.05, and trending towards significance if 0.05 < *P* (or *q*) < 0.1.

### Data availability

The 16S rRNA gene sequences have been submitted to the NCBI Sequence Read Archive SRA and are available under the BioProject ID PRJNA851069.

## Results

### Heat stress induction, animal performance measures, and SCFA concentrations

**Figure 2.**
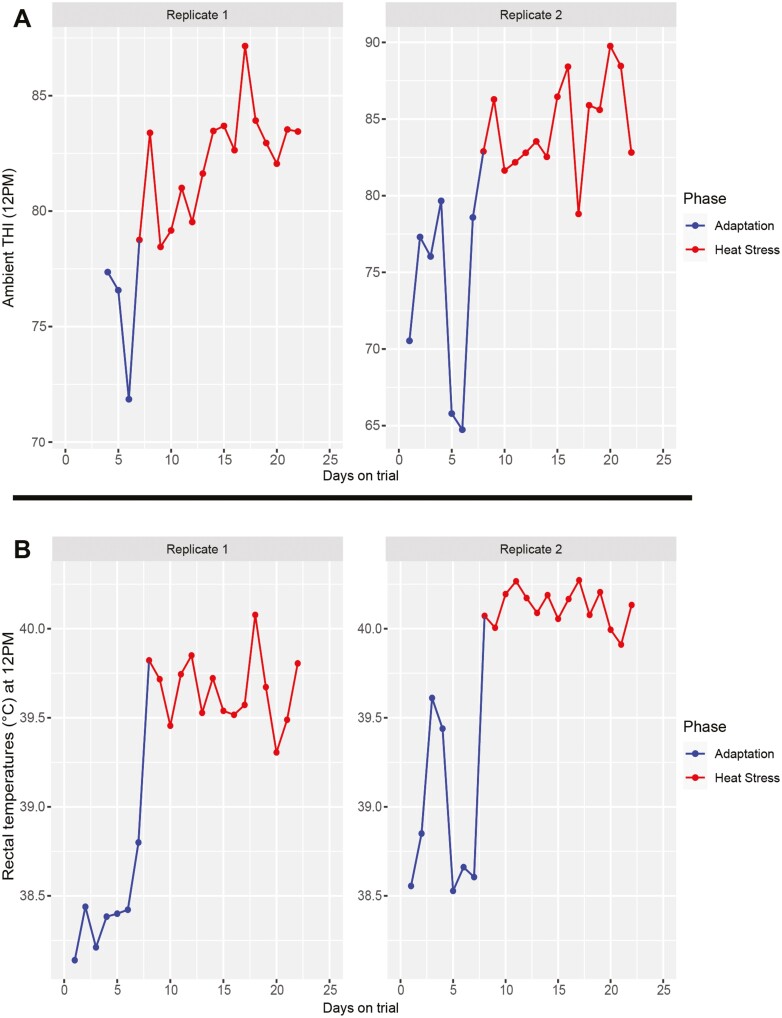
Ambient THI values (A) and average rectal temperatures (B) taken at 1200 h. Average THI was calculated from two temperature/humidity loggers stationed at either side of the barn. Averages of rectal temperatures were calculated across all 10 cows within each replicate.

**Figure 3.**
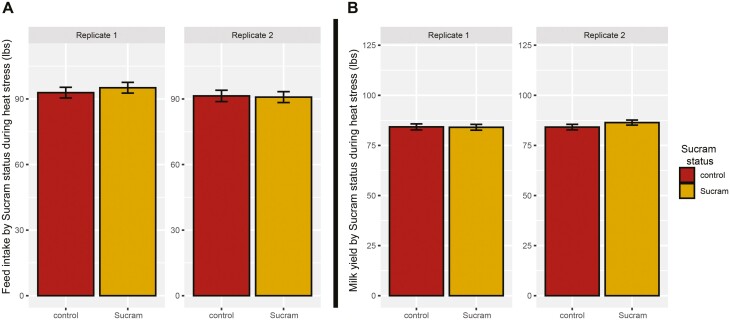
Average (A) animal parameters milk weight yield (MW) and (B) feed intake (FI) by Sucram status, split by replicate. The effect of treatment and replicate were tested using PROC MIXED in SAS according to Eq. (1) in the Methods. Error bars indicate the absolute standard error of the mean. Each measurement (per cow) was corrected by the average adaptation measurements included in the model as a covariate. No significant differences were detected.

Ambient THI and rectal temperatures were successfully increased between 0800 and 1800 h, ensuring heat stress parameters were met. Figure 2 displays average ambient THI at 1200 h during the heat stress phase (ranging between 78 and 89) and average rectal temperatures of all cows at 1200 h (ranging between 39.3 °C and 40.3 °C). No (*P* > 0.05) differences in either milk yield or feed intake were detected between Sucram and control groups regardless of replicate (Figure 3). In addition, no (*P* > 0.05) differences in SCFA concentration were detected between Sucram and control groups for any of the SCFAs tested. Mean SCFA concentrations (mM) are displayed in Supplementary Fig. 1. 

### Rumen content microbial communities

About 1.16 million high-quality sequences were obtained from 40 rumen content samples with an average sequencing depth per sample of 29,202 sequences with a standard deviation of 5,212 sequences. After quality control these sequences were clustered into 4,530 OTUs representing greater than 10 sequences. About 97.4% of the reads were bacterial and 2.6% were archaeal. The 4,530 OTUs were assigned to 19 phyla with *Bacteroidetes*, *Firmicutes*, and *Proteobacteria* being the most abundant and representing 44%, 25%, and 20% of all reads, respectively (Supplementary Fig. 2, Supplementary Table 1). Within the rumen content microbiota dataset OTUs, OTU 1, classified as *Succinivibrionaceae*_UCG-001, was the most abundant and accounted for 14.5% of all reads. It also shared 100% sequence similarity with OTU 1 of the rumen epithelial microbiota dataset. Among the 50 most abundant rumen content OTUs, 26 OTUs were classified within the family *Prevotellaceae*, a family which accounted for 34% of all reads from the rumen content microbiota dataset (Supplementary Fig. 3). A list of the 50 most abundant rumen content microbiota OTUs can be found in Supplementary Table 2.

**Figure 4.**
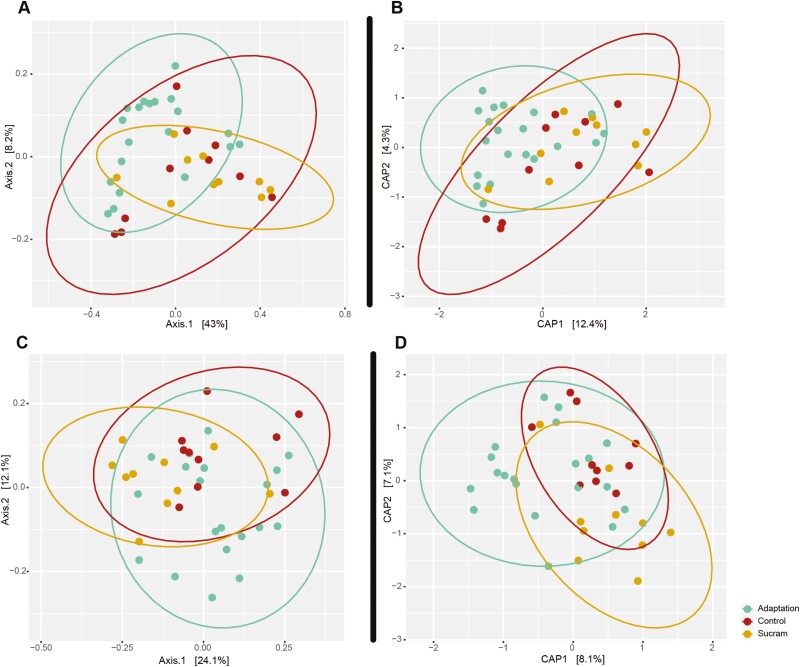
Beta diversity of rumen content microbiota and rumen epithelial microbiota based on Sucram status.Unconstrained (principal coordinates analysis, PCoA) and constrained ordinations (canonical analysis of principle coordinates, CAP) are shown. (A) PCoA for rumen content microbiota, (B) CAP for rumen content microbiota, (C) PCoA for rumen epithelial microbiota (DP CAP for rumen epithelial microbiota). Distances between samples are based on Bray-Curtis dissimilarity measures. The effect of treatment and replicate on microbial beta-diversity were tested using PERMANOVA according to Eq. (1). Differences were detected between the Sucram treatment samples and the adaptation samples within the rumen epithelial microbial communities only. Pairwise contrasts between populations can be found in Supplementary Tables S4 and S8.

No differences were detected when comparing rumen content microbiota alpha diversity measurements between Sucram status experimental groups (Chao, *P* = 0.98, Simpson, *P* = 0.67, Shannon, *P* = 0.69), for the fixed effect of replicate, or the adaptation measure covariate (Supplementary Table 3, Supplementary Fig. 3). Significant differences in rumen content microbiota beta diversity measurements were detected based on Sucram status (*P* = 0.003, *R*^2^ = 0.12), but no difference was detected between replicate (*P* = 0.13) when using PERMANOVA. A difference in beta diversity was detected between the adaptation sampling time point and the Sucram supplemented group after heat stress (*q* = 0.015), however no differences were detected between control and Sucram groups after heat stress (*q* = 0.330). Differences in the adaptation and control groups trended towards significance as well (*q* = 0.093) (Supplementary Table 4). Community clustering of experimental groups can be seen in the PCoA and CAP plots (Figure 4). No differences were detected when comparing individual OTUs, genera, or phyla with regards to Sucram status.

### Rumen epithelium microbiota

About 2.15 million high-quality sequences were obtained from the 40 rumen epithelial samples. The average number of sequences per merged sample was 53,983, with a standard deviation of 10,336 sequences. After quality control and merging, these sequences were clustered into 6,183 OTUs representing more than 10 sequences. Most of the reads (92.3%) were bacterial while 7.6% were archaeal. From the 6,183 OTUs, 21 phyla were identified with *Firmicutes*, *Bacteroidetes*, and *Proteobacteria* being the most abundant, representing 37%, 27%, and 12% of all reads, respectively (Supplementary Table 5, Supplementary Fig. 5). The most abundant OTU within the rumen epithelial microbiota dataset was identified as *Succinivibrionaceae_UCG-001* and accounted for 6.8% of all reads from the rumen epithelial microbiota dataset. OTUs 2, 3, 4, and 5 were classified as *Mogibacterium*, *Desulfobulbus*, *Campylobacter*, and *Methanobrevibacter*, accounting for 2.0%, 1.8%, 1.5%, and 1.4% of all epithelial reads, respectively (Supplementary Fig. 6). A list of the 50 most abundant rumen epithelial microbiota OTUs can be found in Supplementary Table 6.

Unlike the rumen content microbiota, differences in rumen epithelial microbiota alpha diversity measurements between Sucram status experimental groups were detected. We observed no differences in species evenness (Simpson, *P* = 0.54); however, species richness tended to decrease (Chao, *P* = 0.06) and diversity (Shannon, *P* = 0.034) decreased when comparing Sucram to control groups. Additionally, using the adaptation phase as a baseline covariate captured (*P* = 0.03) variation when interpreting differences in Chao species richness between Sucram and control treatment groups (Supplementary Table 7, Supplementary Fig. 4). Differences in beta diversity measurements were also detected between Sucram and control groups (*P* = 0.001, *R*^2^ = 0.12) and between replicates (*P* = 0.008, *R*^2^ = 0.06) when using PERMANOVA (Supplementary Table 8). Visualization of community clustering of experimental groups can be found in the PCoA and CAP plots (Figure 3). Pairwise contrasts revealed differences between adaptation and Sucram (*q* = 0.09) and Sucram and control (*q* = 0.02) as well as a difference trending towards significance between adaptation and control (*q* = 0.056, Supplementary Table 8). No differences were detected when comparing individual rumen epithelial microbiota OTUs, genera, or phyla between levels of Sucram status.

## Discussion

### Synopsis of results

Heat stress is becoming increasingly relevant in livestock agriculture as many regions around the globe are experiencing higher than historical average temperatures (Rossati, 2017). These heat stress events have been shown to greatly impact many aspects of production, including animal performance, health, and efficiency (Silanikove, 2000; St-Pierre et al., 2003; Nonaka et al., 2008; Bishop-Williams et al., 2015; Brown-Brandl, 2018; Menta et al., 2022; Thornton et al., 2022). The results presented in this manuscript describe microbial community structure and composition within two separate rumen microbial communities, rumen content microbiota, and rumen epithelial microbiota, in relation to supplementation of Sucram, a sodium-saccharin based sweetener, during a heat stress event. Additionally, we provide general interpretations of the effects of heat stress on rumen microbial communities, animal performance measures, and SCFA concentrations.

### The effect of heat stress on the rumen microbial communities and host performance

Heat stress has been shown to affect many metabolic processes in dairy cattle beyond the associated reduction in feed intake. Rhoads et al. (2009) determined that dairy cows that were exposed to heat stress had a much greater decrease in milk yield than their pair-fed counterparts (40% and 21%, respectively). Wheelock et al. (2010) continued this line of inquiry to discover a decrease in milk protein, lactose and glucose levels, and lipid mobilization in cows after a heat stress event. Both the decrease in circulating lipids and decrease in protein synthesis during a heat stress event were confirmed in another study by Gao et al. (2017). It is possible that some of these changes are in part related to changes in the microbial communities of the rumen during heat stress. Zhao et al. (2019) found that lactating Holstein cows experiencing heat stress had increased microbial richness in the rumen content microbiota compared to their pair-fed, thermoneutral counterparts. Additionally, they found that the relative abundances of the genera *Spirochaeta*, *Streptococcus*, and *Ruminobacter* in the rumen were increased in heat stress cows and observed along with a decrease in acetic acid concentrations. Cumulatively, the studies suggest the rumen microbiome changes during heat stress in a complex manner that involves more physiological changes than decreased feed intake alone.

This study primarily focused on the effect of supplementing feed with Sucram during heat stress, and consequently the study design confounds the effect of heat stress with time. However, despite the limitations of the design, many of the observations in regards to heat stress and microbial community composition are worth noting. Most notably, differences in rumen epithelial microbiota composition were detected between adaptation samples and both Sucram and control treatment groups (*P* < 0.001, *P* < 0.01). This effect was not observed in the rumen content microbiota, however, which may indicate differential effects of heat stress between these rumen microbial communities. No individual OTUs were found to shift due to heat stress in either community, suggesting that the observed differences in community composition are driven by minor changes across a wide range of microbial species rather than a dramatic shift in individual taxa. Importantly, although there are a limited number of studies analyzing the rumen content microbiota in response to heat stress, this is the first work to observe an effect of heat stress on the rumen epithelial microbiota. To reiterate, the rumen epithelial microbiota is involved in many host-relevant metabolic functions including oxygen scavenging, nitrogen cycling, and ­barrier function (Cheng et al., 1979; Mann et al., 2018). Future work designed to examine the rumen epithelial microbiota changes strictly caused by heat stress would be valuable to better understand differences between how the rumen content microbiota and rumen epithelial microbiota respond to heat stress. The paucity of information regarding the rumen epithelial microbiota response to heat stress and its effect on livestock production demonstrates the value of additional future research to be conducted in this area.

### The effect of Sucram supplementation on the rumen microbial communities

Previous studies focusing on the effect of Sucram supplementation have reported changes in microbial communities of mucosal bacteria within the gastrointestinal tract in suckling piglets and mice. Piglets supplemented with 0.015% w/w Sucram demonstrated changes in the abundance of several bacterial taxa, including increases in *Lactobacillus* and *Helicobacter* sp. and decreases in *Campylobacter*, *Ruminoccocaceae*, and *Veillonellaceae* (Daly et al., 2014, 2016; Suez et al., 2014; Kelly et al., 2017). In addition, a recent study applied a combination of Sucram and capsaicin and showed a beneficial effect in pigs under heat stress (Kroscher et al., 2022). A reduction of *Ruminococcus* was also documented in a study analyzing the effect of 0.3 mg/mL saccharin, the major component of Sucram, on inflammatory molecules and gut dysbiosis in mice (Bian et al., 2017).

Although the current study did not detect significant differences in specific taxa or changes in feed intake, SCFA production, or milk yield, it did reveal a general shift in microbial community structure and composition. It is hard to determine the reason behind both the more pronounced changes occurring in the rumen epithelial microbiota when cows were experiencing heat stress and the reduction in rumen epithelial microbiota species diversity in cows supplemented with Sucram. Other non-nutritive sweeteners have been shown to increase reactive oxygen species which may eliminate less oxygen-tolerant microorganisms, possibly reducing species richness within a system, but this effect did not occur when saccharin was tested (Yu et al., 2021). This same study found that several sweeteners, including saccharin, increased conjugative plasmid transfer, which may make certain receiving microorganisms more resilient to heat stress. Previous studies have shown that heat-resistance genes can be transferred on plasmids, conferring heat-stress to a number of different microbial species (Yu et al., 2021). However, much higher concentrations of saccharin were required to demonstrate this effect comparatively to the other sweeteners. Notably, to test the effect of saccharin on plasmid conjugation, Yu and colleagues (Yu et al., 2021) utilized saccharin concentrations ranging from 0.3 mg/L to 300 mg/L, whereas the concentration of Sucram used in the current study was approximately 13 mg/L, assuming 2 grams of Sucram into 151 L of rumen content. Thus, it is possible that the effect size observed in this work might have been greater if higher levels of Sucram were used. Although so far reported only in pigs, a combination of Sucram with other feed additives such as capsaicin might be an additional alternative for future research (Kroscher et al., 2022). Additionally, in vitro culturing assays like those conducted by Yu et al. (2021) or gene expression studies may provide additional data about the microbial response to Sucram.

### Potential of rumen microbial communities to degrade saccharin

A recent study provided evidence that certain bacteria within wastewater degrade saccharin-based artificial sweeteners, raising the possibility that artificial sweeteners may not be noncaloric to the host if they are converted into compounds by the microbiota that can then be metabolized by the host (Deng et al., 2019). Two studies have shown that saccharin-based artificial sweeteners increased concentrations of lactic, propionic, and acetic acids within the porcine and murine gastrointestinal tract, further suggesting a potential bacterial metabolism of saccharin (Suez et al., 2014; Daly et al., 2016). In an effort to discover the genes required to metabolize saccharin, Deng et al. (2019) analyzed the microbial community composition and genetic content of activated biowaste incubated with saccharin. They found that members of the class *Betaproteobacteria* were enriched in the saccharin-supplemented systems. Additionally, they suggest several metabolic pathways that may be involved in saccharin degradation. The large metabolic capacity, controlled temperatures, and anaerobic nature of the rumen environment makes it a promising location to screen for organisms containing novel chemical breakdown pathways (Allison et al., 1985, 1992; Cook et al., 1994). Further research using a metagenomic or metatranscriptomic approach might indicate specific pathways involved in saccharin/Sucram metabolism.

Suez et al. (2014) performed metagenomic sequencing of murine fecal samples after supplementation of commercial saccharin sweeteners such as Sucram and pure saccharin. They found that glycan degradation genes were more abundant in the saccharin-supplemented group compared to the control. Additionally, higher concentrations of SCFAs were observed in the supplemented group compared to the control. Although no differences in SCFA concentrations were observed in the present study, the metabolic pathways for the synthesis of SCFAs are encoded in the genomes of members of the rumen content microbiota and rumen epithelial microbiota. As the rumen content microbiota was less responsive to Sucram supplementation and is the primary site of glycan degradation to SCFAs, this may explain the observed lack of change in SCFA concentrations.

## Conclusion

The work presented here aimed to test the effect of the saccharin-based sweetener Sucram on rumen microbial communities in the context of heat stress. We recorded animal performance measures and SCFA concentration measurements with the intention to link microbial effects to shifts in animal performance. We determined that Sucram had impacts on rumen microbial alpha and beta diversity measurements, resulting in a decrease in overall community diversity and whole community differences. These changes were not driven by any specific taxa, nor did they coincide with changes in animal performance. This may indicate that functional redundancy is present within existing rumen microbial communities. Further, the magnitude of change observed in rumen epithelial microbiota was greater than was observed in rumen content microbiota. This study suggests that Sucram does have an effect on rumen microbial populations. Future work will need to focus on more functional aspects of the effect of Sucram supplementation in regards to both microbiota and subsequent host performance, with additional focus on the rumen epithelial microbiota.

## Supplementary Material

skac403_suppl_Supplementary_Figure_S1

skac403_suppl_Supplementary_Figure_S2

skac403_suppl_Supplementary_Figure_S3

skac403_suppl_Supplementary_Figure_S4

skac403_suppl_Supplementary_Figure_S5

skac403_suppl_Supplementary_Figure_S6

skac403_suppl_Supplementary_Table_S1

skac403_suppl_Supplementary_Table_S2

skac403_suppl_Supplementary_Table_S3

skac403_suppl_Supplementary_Table_S4

skac403_suppl_Supplementary_Table_S5

skac403_suppl_Supplementary_Table_S6

skac403_suppl_Supplementary_Table_S7

skac403_suppl_Supplementary_Table_S8

## Abbreviations

ADG: average daily gain
DMI: dry matter intake
GI: gastrointestinal
OTU: operational taxonomic unit
PBS: phosphate buffered saline
SCFA: short chain fatty acids
THI: temperature humidity index
