# Supplementary figures and images for: Influence of a sodium-saccharin sweetener on the rumen content and rumen epithelium microbiota in dairy cattle during heat stress
Source: J Anim Sci. 2022 Dec 13;101:skac403. doi: 10.1093/jas/skac403 (PMC9838801; doi:10.1093/jas/skac403)

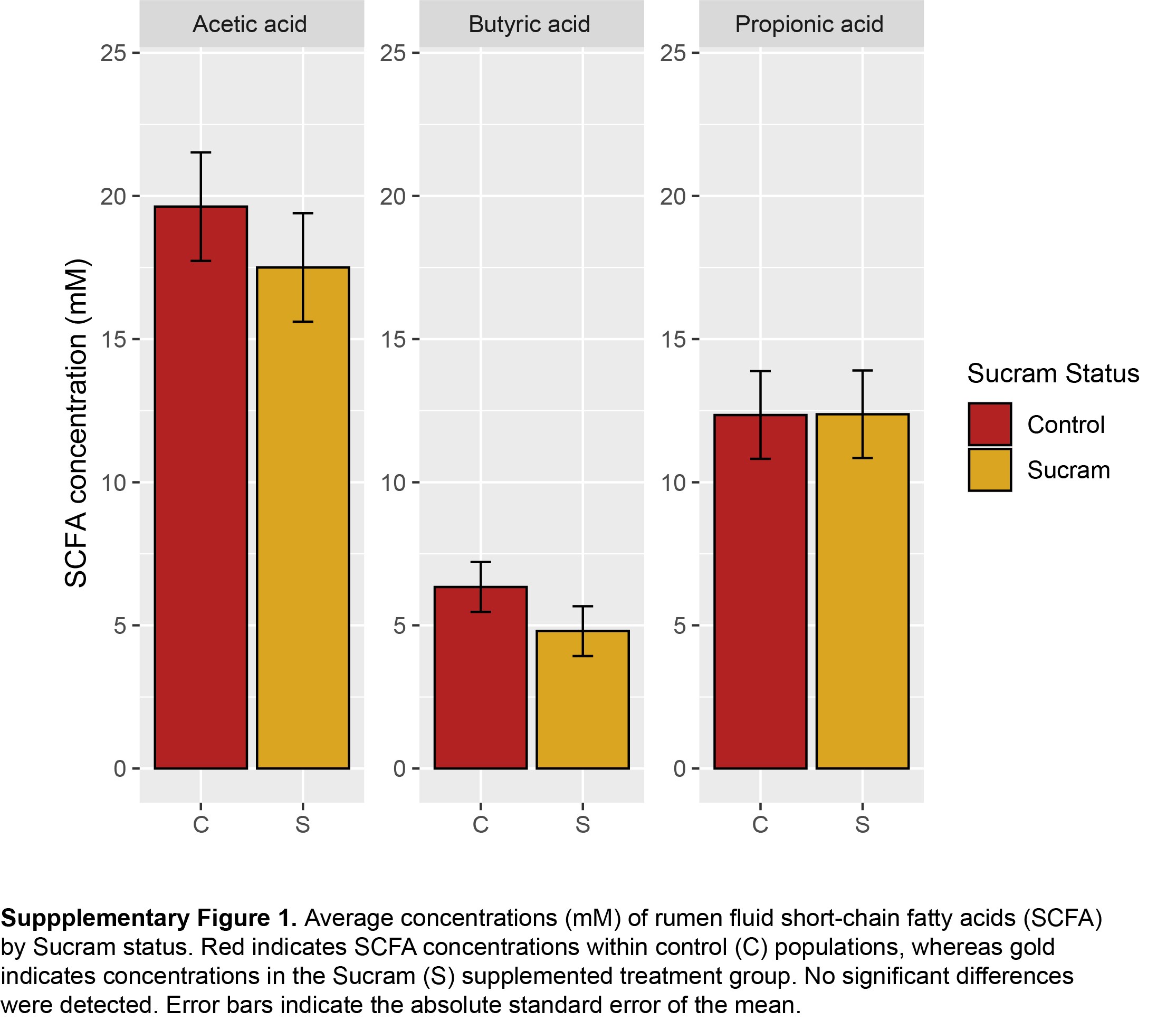

Supplement: skac403_suppl_Supplementary_Figure_S1 [file skac403_suppl_supplementary_figure_s1.jpeg]

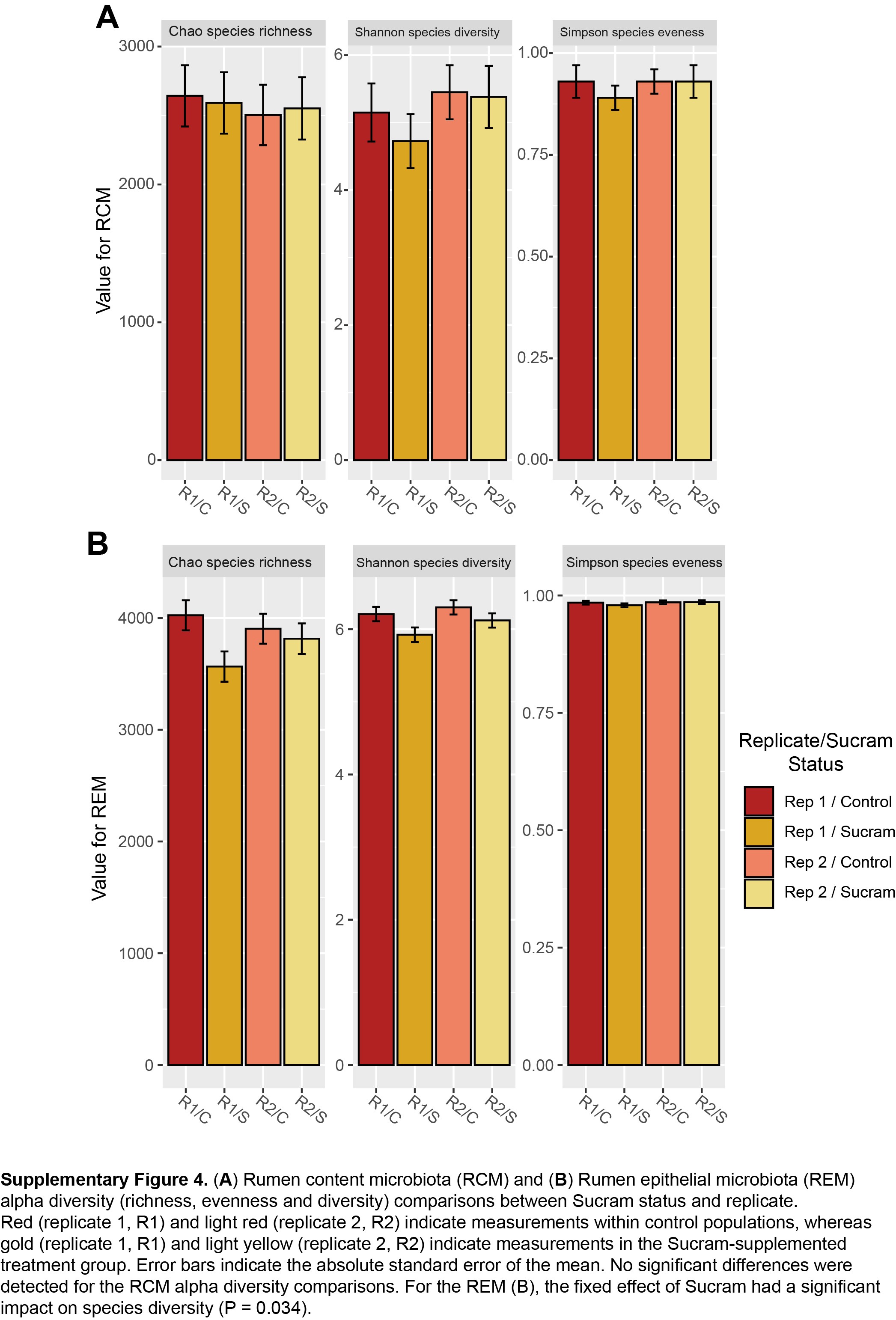

Supplement: skac403_suppl_Supplementary_Figure_S4 [file skac403_suppl_supplementary_figure_s4.jpeg]
