# Supplementary material for: Influence of a sodium-saccharin sweetener on the rumen content and rumen epithelium microbiota in dairy cattle during heat stress
Source: J Anim Sci. 2022 Dec 13;101:skac403. doi: 10.1093/jas/skac403 (PMC9838801; doi:10.1093/jas/skac403)
Supplement: skac403_suppl_Supplementary_Figure_S6 [file skac403_suppl_supplementary_figure_s6.docx]

**
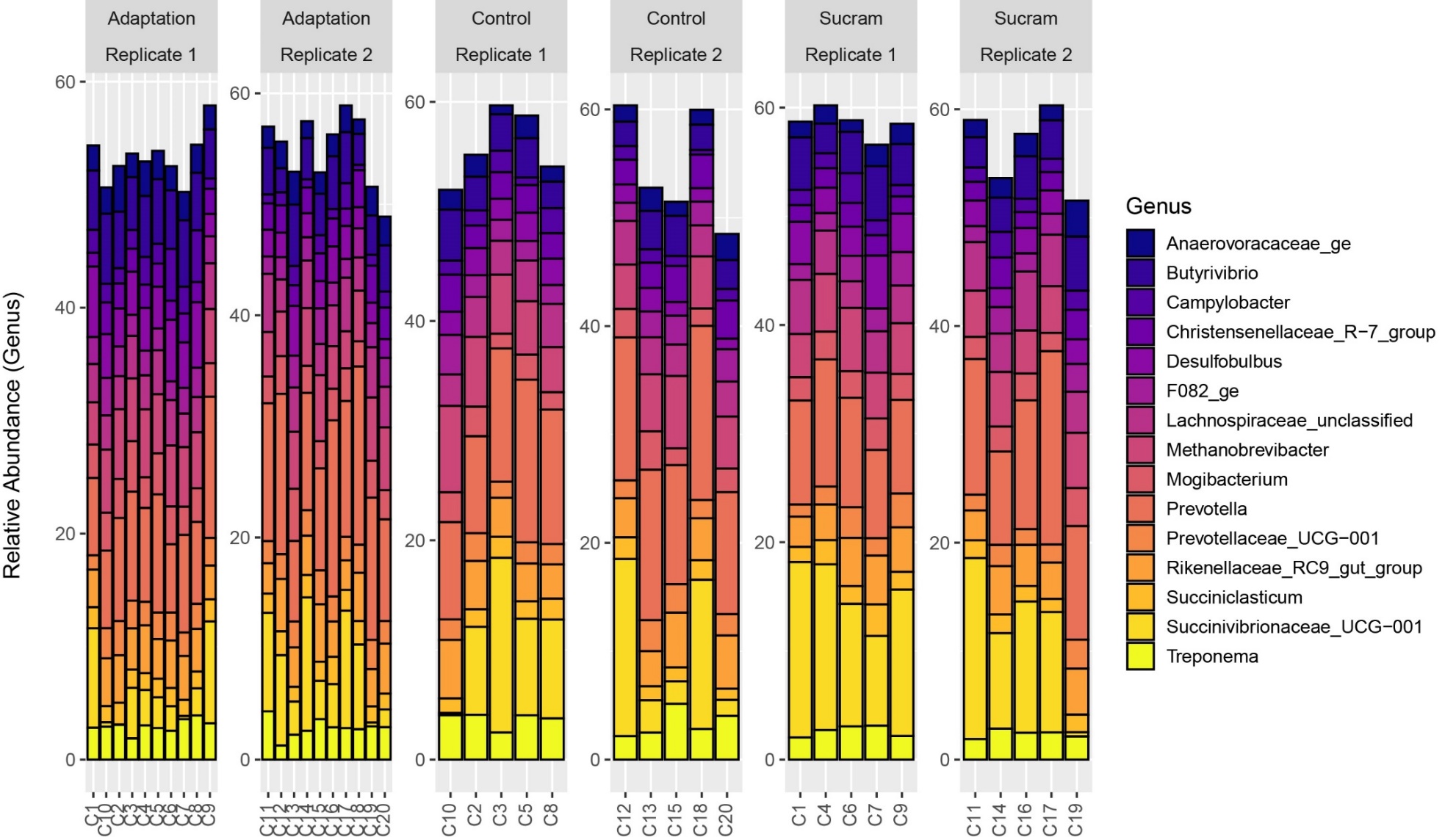
**

**Supplementary Figure 6. Unadjusted relative abundance of the 15 most abundant genera in rumen epithelium microbiota (REM).** Separation between Sucram status and replicate is shown. Cows used in the experiment (C1-C20) are shown on the x axis.
