# Supplementary material for: Influence of a sodium-saccharin sweetener on the rumen content and rumen epithelium microbiota in dairy cattle during heat stress
Source: J Anim Sci. 2022 Dec 13;101:skac403. doi: 10.1093/jas/skac403 (PMC9838801; doi:10.1093/jas/skac403)
Supplement: skac403_suppl_Supplementary_Table_S1 [file skac403_suppl_supplementary_table_s1.docx]

**Supplementary Table 1.** **Relative abundance of the ten most abundant RCM^1^ bacterial and archaeal phyla based on 16S rRNA gene amplicon sequencing^2^.**

| **Phyla** | **Relative abundance (%)** |
| --- | --- |
| *Firmicutes* | 37.39 |
| *Bacteroidota* | 27.27 |
| *Proteobacteria* | 12.04 |
| *Euryarchaeota* | 6.25 |
| *Desulfobacterota* | 3.63 |
| *Spirochaetota* | 3.33 |
| *Campilobacterota* | 1.62 |
| *Fibrobacterota* | 1.35 |
| *Thermoplasmatota* | 1.28 |
| *Synergistota* | 1.21 |

**^1^**RCM - Rumen content microbiota

**^2^**Paired end 16S rRNA gene amplicon sequencing was done using the Illumina MiSeq platform. Sequence data was analyzed using Mothur v1.43.0 and taxonomic assignment was completed using the Silva reference database v138
