# Supplementary material for: Influence of a sodium-saccharin sweetener on the rumen content and rumen epithelium microbiota in dairy cattle during heat stress
Source: J Anim Sci. 2022 Dec 13;101:skac403. doi: 10.1093/jas/skac403 (PMC9838801; doi:10.1093/jas/skac403)
Supplement: skac403_suppl_Supplementary_Table_S2 [file skac403_suppl_supplementary_table_s2.docx]

**Supplementary Table 2.** **The 50 most abundant OTUs^1^ across all RCM^2^ samples used in this study based on 16S rRNA gene amplicon sequencing^3^.**

| **OTU** | **Relative abundance (%)** | **Phylum** | **Taxonomy (Silva v138)** |
| --- | --- | --- | --- |
| OTU 1 | 14.50 | *Proteobacteria* | *Succinivibrionaceae*_UCG-001 |
| OTU 2 | 3.58 | *Proteobacteria* | *Succinivibrionaceae*_UCG-002 |
| OTU 3 | 3.34 | *Bacteroidota* | *Prevotella* |
| OTU 4 | 1.55 | *Bacteroidota* | *Prevotellaceae*_UCG-004 |
| OTU 5 | 1.20 | *Bacteroidota* | *Prevotella* |
| OTU 6 | 0.97 | *Euryarchaeota* | *Methanobrevibacter* |
| OTU 7 | 0.79 | *Firmicutes* | *Lachnospiraceae*_NK3A20 |
| OTU 8 | 0.74 | *Firmicutes* | *Succiniclasticum* |
| OTU 9 | 0.71 | *Bacteroidota* | *Prevotella* |
| OTU 10 | 0.63 | *Firmicutes* | *Christensenellaceae*_R-7 |
| OTU 11 | 0.63 | *Bacteroidota* | p-251-o5 |
| OTU 12 | 0.61 | *Bacteroidota* | *Rikenellaceae*_RC9_gut |
| OTU 13 | 0.58 | *Firmicutes* | unclassified *Ruminococcaceae* |
| OTU 14 | 0.58 | *Bacteroidota* | *Prevotella* |
| OTU 15 | 0.53 | *Bacteroidota* | *Prevotella* |
| OTU 16 | 0.52 | *Bacteroidota* | *Prevotella* |
| OTU 17 | 0.47 | *Bacteroidota* | *Prevotella* |
| OTU 18 | 0.46 | *Bacteroidota* | *Prevotella* |
| OTU 19 | 0.44 | *Bacteroidota* | *Prevotella* |
| OTU 20 | 0.44 | *Bacteroidota* | *Prevotella* |
| OTU 21 | 0.42 | *Firmicutes* | *Oscillospiraceae* NK4A214 |
| OTU 22 | 0.40 | *Bacteroidota* | *Prevotella* |
| OTU 23 | 0.38 | *Bacteroidota* | *Prevotella* |
| OTU 24 | 0.38 | *Bacteroidota* | *Prevotella* |
| OTU 25 | 0.38 | *Patescibacteria* | *Absconditabacteriales*_(SR1) |
| OTU 26 | 0.37 | *Euryarchaeota* | *Methanobrevibacter* |
| OTU 27 | 0.36 | *Bacteroidota* | *Prevotella* |
| OTU 28 | 0.35 | *Firmicutes* | *Sharpea* |
| OTU 29 | 0.35 | *Bacteroidota* | *Prevotellaceae*_YAB2003 |
| OTU 30 | 0.35 | *Bacteroidota* | *Prevotella* |
| OTU 31 | 0.32 | *Spirochaetota* | *Treponema* |
| OTU 32 | 0.32 | *Proteobacteria* | *Ruminobacter* |
| OTU 33 | 0.31 | *Bacteroidota* | *Bacteroidales*_RF16 |
| OTU 34 | 0.30 | *Proteobacteria* | *Ruminobacter* |
| OTU 35 | 0.30 | *Firmicutes* | *Ruminococcus* |
| OTU 36 | 0.30 | *Firmicutes* | NK4A214 |
| OTU 37 | 0.29 | *Bacteroidota* | F082 |
| OTU 38 | 0.29 | *Euryarchaeota* | *Methanobrevibacter* |
| OTU 39 | 0.29 | *Bacteroidota* | *Prevotella* |
| OTU 40 | 0.29 | *Euryarchaeota* | *Methanobrevibacter* |
| OTU 41 | 0.27 | *Spirochaetota* | *Treponema* |
| OTU 42 | 0.27 | *Bacteroidota* | *Prevotella* |
| OTU 43 | 0.26 | *Bacteroidota* | *Prevotellaceae*_UCG-004 |
| OTU 44 | 0.24 | *Bacteroidota* | Uncultured |
| OTU 45 | 0.24 | *Bacteroidota* | *Prevotella* |
| OTU 46 | 0.24 | *Bacteroidota* | *Prevotella* |
| OTU 47 | 0.24 | *Bacteroidota* | *Prevotella* |
| OTU 48 | 0.24 | *Bacteroidota* | *Prevotella* |
| OTU 49 | 0.23 | *Bacteroidota* | unclassified *Prevotellaceae* |
| OTU 50 | 0.23 | *Bacteroidota* | *Prevotella* |

**^1^**OTU - Operational taxonomic unit

**^2^**RCM - Rumen content microbiota

**^3^**Paired end 16S rRNA gene amplicon sequencing was done using the Illumina MiSeq platform. Sequence data was analyzed using Mothur v1.43.0 and taxonomic assignment was completed using the Silva reference database v138
