# Supplementary material for: Influence of a sodium-saccharin sweetener on the rumen content and rumen epithelium microbiota in dairy cattle during heat stress
Source: J Anim Sci. 2022 Dec 13;101:skac403. doi: 10.1093/jas/skac403 (PMC9838801; doi:10.1093/jas/skac403)
Supplement: skac403_suppl_Supplementary_Table_S3 [file skac403_suppl_supplementary_table_s3.docx]

**Supplementary Table 3.** **Alpha diversity comparison results when comparing RCM^1^ by Sucram status adjusted by the adaptation phase**.

| **Chao species richness** | | | | | | |
| --- | --- | --- | --- | --- | --- | --- |
| Type 3 Tests of Fixed Effects | | | | | | |
| **Effect** | **Num DF^2^** | **Den DF^3^** | | **F Value** | | ***P*-value** |
| Sucram^®^ status | 1 | 15 | | 0 | | 0.99 |
| Replicate | 1 | 15 | | 0.14 | | 0.72 |
| Sucram^®^ status*Replicate | 1 | 15 | | 0.05 | | 0.82 |
| adaptation | 1 | 15 | | 0.85 | | 0.37 |
|  | | | | | | |
| **Simpson evenness** | | | | | | |
| Type 3 Tests of Fixed Effects | | | | | | |
| **Effect** | **Num DF^2^** | **Den DF^3^** | | **F Value** | | ***P*-value** |
| Sucram^®^ status | 1 | 15 | | 0.26 | | 0.62 |
| Replicate | 1 | 15 | | 0.37 | | 0.55 |
| Sucram^®^ status*Replicate | 1 | 15 | | 0.19 | | 0.67 |
| adaptation | 1 | 15 | | 1.50 | | 0.24 |
|  | | | | | | |
| **Shannon diversity** | | | | | | |
| Type 3 Tests of Fixed Effects | | | | | | |
| **Effect** | **Num DF^2^** | | **Den DF^3^** | | **F Value** | ***P*-value** |
| Sucram^®^ Status | 1 | | 15 | | 0.30 | 0.59 |
| Replicate | 1 | | 15 | | 1.13 | 0.30 |
| Sucram^®^ status*Replicate | 1 | | 15 | | 0.19 | 0.67 |
| adaptation | 1 | | 15 | | 1.70 | 0.21 |

**^1^**RCM - Rumen content microbiota

**^2^**Num DF - Numerator degrees of freedom

**^3^**Den DF - Denominator degrees of freedom
