# Supplementary material for: Influence of a sodium-saccharin sweetener on the rumen content and rumen epithelium microbiota in dairy cattle during heat stress
Source: J Anim Sci. 2022 Dec 13;101:skac403. doi: 10.1093/jas/skac403 (PMC9838801; doi:10.1093/jas/skac403)
Supplement: skac403_suppl_Supplementary_Table_S4 [file skac403_suppl_supplementary_table_s4.docx]

**Supplementary Table 4.** **PERMANOVA, BetaDisperser and pairwise PERMANOVA results when using Eq. 1 to determine differences in RCM^1^ community composition due to Sucram status and replicate during heat stress and compared to adaptation.**

| **PERMANOVA** | | | | | | | |
| --- | --- | --- | --- | --- | --- | --- | --- |
| Command: adonis2(formula = data_bray ~ Sucram_status + Replicate + Sucram_status * Replicate, data = data_sub_type_df) | | | | | | | |
| **Effect** | **DF^2^** | **Sum sq^3^** | **R2^5^** | **F** | | | ***P*-value** |
| Sucram^®^ status | 2 | 0.61 | 0.12 | 2.46 | | | 0.01 |
| Replicate | 1 | 0.20 | 0.04 | 1.59 | | | 0.130 |
| Sucram^®^ status*Replicate | 2 | 0.21 | 0.04 | 0.86 | | | 0.583 |
| Residual | 34 | 4.19 | 0.81 |  | | |  |
| Total | 39 | 5.21 |  |  | | |  |
| **Beta Disperser** | | | | | | | |
| Command: betadisper(data_bray, data_sub_type_df$sucram_adjust) | | | | | | | |
|  | **DF^2^** | **Sum Sq^3^** | **Mean Sq^4^** | | | **F** | ***P*-value** |
| Groups | 2 | 0.008 | 0.004 | | 0.60 | | 0.55 |
| Residuals | 37 | 0.249 | 0.006 | |  | |  |
| **Pairwise PERMANOVA** | | | | | | | |
| Command: pairwise.adonis(data_bray, sample_data(data_sub_type)$sucram_adjust, perm = 10000) | | | | | | | |
| **pairs** | **DF^2^** | **Sum Sq^3^** | **F.Model** | | **R2^5^** | | ***P*-value** |
| Adaptation vs Control | 1 | 0.22 | 1.717 | | 0.058 | | 0.093 |
| Adaptation vs Sucram | 1 | 0.51 | 4.33 | | 0.134 | | 0.002 |
| Control vs Sucram | 1 | 0.13 | 0.969 | | 0.051 | | 0.330 |

**^1^**RCM - Rumen content microbiota

**^2^**DF - Degrees of freedom

**^3^**Sum Sq - Sum of squares

**^4^**Mean Sq - Mean sum of squares

**^5^**R2 - Coeffficient of determination
