# Supplementary material for: Influence of a sodium-saccharin sweetener on the rumen content and rumen epithelium microbiota in dairy cattle during heat stress
Source: J Anim Sci. 2022 Dec 13;101:skac403. doi: 10.1093/jas/skac403 (PMC9838801; doi:10.1093/jas/skac403)
Supplement: skac403_suppl_Supplementary_Table_S7 [file skac403_suppl_supplementary_table_s7.docx]

**Supplementary Table 7.** **Alpha diversity comparison results when comparing REM^1^ samples separated by Sucram status adjusted by the adaptation phase**.

| **Chao species richness** | | | | |
| --- | --- | --- | --- | --- |
| Type 3 Tests of Fixed Effects | | | | |
| **Effect** | **Num DF** | **Den DF** | **F Value** | ***P*-value** |
| Sucram^®^ status | 1 | 15 | 4.16 | 0.06 |
| Replicate | 1 | 15 | 0.22 | 0.65 |
| Sucram^®^ status*Replicate | 1 | 15 | 1.86 | 0.19 |
| Adaptation | 1 | 15 | 5.78 | 0.03 |
|  |  |  |  |  |
| **Simpson evenness** | | | | |
| Type 3 Tests of Fixed Effects | | | | |
| **Effect** | **Num DF** | **Den DF** | **F Value** | ***P*-value** |
| Sucram^®^ status | 1 | 15 | 0.38 | 0.55 |
| Replicate | 1 | 15 | 0.88 | 0.36 |
| Sucram^®^ status*Replicate | 1 | 15 | 0.58 | 0.46 |
| Adaptation | 1 | 15 | 3.23 | 0.09 |
|  |  |  |  |  |
| **Shannon diversity** | | | | |
| Type 3 Tests of Fixed Effects | | | | |
| **Effect** | **Num DF** | **Den DF** | **F Value** | ***P*-value** |
| Sucram^®^ status | 1 | 15 | 5.45 | 0.034 |
| Replicate | 1 | 15 | 2.11 | 0.167 |
| Sucram^®^ status*Replicate | 1 | 15 | 0.28 | 0.602 |
| Adaptation | 1 | 15 | 4.35 | 0.055 |

**^1^**REM - Rumen epithelium microbiota

**^2^**Num DF - Numerator degrees of freedom

**^3^**Den DF - Denominator degrees of freedom
