# Supplementary material for: Influence of a sodium-saccharin sweetener on the rumen content and rumen epithelium microbiota in dairy cattle during heat stress
Source: J Anim Sci. 2022 Dec 13;101:skac403. doi: 10.1093/jas/skac403 (PMC9838801; doi:10.1093/jas/skac403)
Supplement: skac403_suppl_Supplementary_Table_S8 [file skac403_suppl_supplementary_table_s8.docx]

**Supplementary Table 8.** **PERMANOVA, BetaDisperser and pairwise PERMANOVA results when using Eq. 1 to determine differences in REM^1^ community composition due to Sucram status and replicate during heat stress and compared to adaptation.**

| **PERMANOVA** | | | | | | | |
| --- | --- | --- | --- | --- | --- | --- | --- |
| Command: adonis2(formula = data_bray ~ Sucram_status + Replicate + Sucram_status * Replicate, data = data_sub_type_df) | | | | | | | |
| **Effect** | **DF^2^** | **Sum Sq^3^** | **R2^5^** | **F** | | | ***P*-value** |
| Sucram^®^ status | 2 | 0.5033 | 0.12 | 2.62 | | | 0.001 |
| Replicate | 1 | 0.2571 | 0.06 | 2.68 | | | 0.008 |
| Sucram^®^ status*Replicate | 2 | 0.1552 | 0.04 | 0.81 | | | 0.764 |
| Residual | 34 | 3.2601 | 0.78 |  | | |  |
| Total | 39 | 4.1756 | 1 |  | | |  |
| **Beta Disperser** | | | | | | | |
| Command: betadisper(data_bray, data_sub_type_df$sucram_adjust) | | | | | | | |
|  | **DF^2^** | **Sum Sq^3^** | **Mean Sq^4^** | | | **F** | ***P*-value** |
| Groups | 2 | 0.007 | 0.003 | | 1.33 | | 0.258 |
| Residuals | 37 | 0.093 | 0.003 | |  | |  |
| **Pairwise PERMANOVA** | | | | | | | |
| Command: pairwise.adonis(data_bray, sample_data(data_sub_type)$sucram_adjust, perm = 10000) | | | | | | | |
| **pairs** | **DF^2^** | **Sum Sq^3^** | **F.Model** | | **R2^5^** | | **p.value** |
| Adaptation vs Control | 1 | 0.306 | 3.096 | | 0.100 | | 0.003 |
| Adaptation vs Sucram | 1 | 0.199 | 1.947 | | 0.065 | | 0.019 |
| Control vs Sucram | 1 | 0.249 | 2.611 | | 0.127 | | 0.008 |

**^1^**REM - Rumen epithelium microbiota

**^2^**DF - Degrees of freedom

**^3^**Sum Sq - Sum of squares

**^4^**Mean Sq - Mean sum of squares

**^5^**R2 - Coeffficient of determination
